# Beliefs about hypertension among primary health care workers and clients in Nigeria: A qualitative study

**DOI:** 10.1371/journal.pone.0209334

**Published:** 2018-12-19

**Authors:** James Tosin Akinlua, Richard Meakin, Ibrahim Bashir, Nick Freemantle

**Affiliations:** 1 Department of Primary Care and Population Health, University College London Medical School (Royal Free Campus), London, United Kingdom; 2 Department of Public health, State House Medical Centre, PMB, Aso Rock Abuja, Nigeria; New York University School of Medicine, UNITED STATES

## Abstract

**Objective:**

The main objective of the study was to elicit beliefs about hypertension among Nigerian Primary Health Care clients and workers.

**Background:**

In many regions of Nigeria, the primary health care facility is usually the only source of formal health care available. Since hypertension is a chronic condition that requires lifelong life style modification and drug treatment, it is important to understand the context and background to the condition through the beliefs and perceptions among both lay persons and health care providers who manage the condition.

**Setting:**

Rural and urban primary health care facilities in the Federal Capital Territory Nigeria.

**Participants:**

A total of Eighty-one (81) primary health care clients and workers participated in the study.

**Methods and outcome measure:**

A qualitative research using interviews, focus group discussions and reflective work as sources of data collection. The outcome measures were emerging themes from thematic framework analysis.

**Results:**

There were four themes that summarize beliefs of both PHC workers and clients namely: (1) Meaning of hypertension (2) causes of hypertension, (3) Consequences of hypertension (4) Perceptions of treatment, one additional distinct theme was elicited among PHC workers “contextual explanation”. However, under each of the shared four themes, there were both similarities and differences in beliefs expressed between PHC workers and clients.

**Conclusions:**

This study highlights important similarities and differences in beliefs about hypertension among primary health care clients and primary health care workers that have significant implications for management of hypertension in primary care settings in Nigeria.

## Introduction

Hypertension is a global public health problem [1]. Currently, it affects about a billion people worldwide[2], but is projected to affect 1.56 billion people and result in 7 million deaths annually by 2025[3].

According to a recent WHO report, it is estimated that the African continent has the highest prevalence rate of hypertension, 46%, among adults aged 25 years and above compared to the Americas, East Mediterranean, Europe, South East Asia and Western Pacific with 35%, 41%, 41%, 37% and 38% respectively [2, 3].

Nigeria with a population of about 180 million bears a substantial amount of this burden [3, 4]. A recent study estimated the crude prevalence of hypertension in Nigeria to be between 2.1% to 47.2% depending on the blood pressure cut-off point, setting, sex and ethnic group being assessed [5]. In many regions of Nigeria, the government owned primary health care (PHC) facility is usually the only source of formal health care available. However, many clients who use PHC facilities may have hypertension or significant cardiovascular disease risk that goes undetected and untreated.

Since, hypertension is a chronic condition that requires lifestyle modification and drug treatment, it is important to understand the context and background to the condition through the beliefs and perceptions among both lay persons(known hypertensives and those not known to be hypertensive) and health care providers. This is particularly pertinent as research has shown that there are differences in perceptions and beliefs known as explanatory models (EM) between health care providers and clients [4, 6]. These differences in EMs are an important determinant of health outcomes and also help inform appropriate management programs [4, 6].

In Nigeria, most of the existing literatures [7–14] in Nigeria on beliefs about hypertension have been conducted in the western part of Nigeria and more than 80% of the study participants are from the Yoruba ethnic group. Given that there are over 350 ethnic groups in Nigeria and there are many other parts of Nigeria with distinct languages and ethnic beliefs, there is need to conduct similar studies in other locations with different mix of ethnicities in order to elicit possible differences or similarities.

Previous studies have focused on the beliefs of patients diagnosed with hypertension [7–14]. However, the beliefs about this condition among people in the community who have not been diagnosed with hypertension may be different as process of diagnosis and treatment may influence their beliefs and understanding. In order to develop preventative programmes it is important to understand the beliefs and understanding held by the wider community.

Furthermore, some studies were conducted in a tertiary health care setting and among insured patients. Tertiary health care settings are quite unique in that many patients who use these facilities might already have cardiovascular complications. Moreover availability of health insurance is still very limited across Nigeria. Very few studies have examined majority of non-insured Nigerians who use government owned Primary health care facilities.

In addition, there is paucity of studies in Nigeria that have examined the specific differences in belief of healthcare workers and clients about hypertension and how it might help inform management of the condition.

In order to explore these areas we undertook a qualitative study among both hypertensive clients, clients not known to be hypertensive and healthcare workers in community-based government owned primary health care facilities in The Federal Capital Territory, Nigeria.

## Methods

The study design was qualitative study using 2 study designs—semi-structured interviews and focus group discussions (FGD) to examine the beliefs of primary health care clients and primary health care workers. This allowed triangulation of the results.

### Setting

The study took place in the Federal Capital Territory (FCT) Abuja, Nigeria. The predominant indigenous inhabitants of Abuja are the Gbagyi (Gwari) ethnic group. However, since the FCT became the capital of Nigeria in 1991, there has been influx of other ethnic groups into FCT thereby making the setting cosmopolitan with members of most ethnic groups residing there. The FCT has six (6) area councils namely: Abaji, Abuja Municipal Area Council, Bwari, Gwagwalada, Kuje and Kwali [15].

The study population area is a mixture of rural and urban settlements and primary health care facilities are fairly evenly distributed across the urban and rural areas.

In Nigeria, there are different categories of primary care facilities depending on the complexities of services available. These include Primary Health Centres, Primary Health Clinics and Health Posts. Among these primary health care facilities, the Primary Health Centres (PHCs)(formerly known as comprehensive PHCs) which offers the most comprehensive services were the setting for this study. There are ten (10) electoral wards per area council and each electoral ward have at least one comprehensive PHC facility making a total of at least sixty (60) PHCs facilities in the six (6) area councils of the FCT evenly distributed across urban and rural areas (i.e. 30 rural and 30 urban PHC facilities in FCT).

### Ethics

Ethical approval was initially obtained from the University College London Ethics Committee (Project ID number 7811/002), thereafter from the Nigerian FCT Health Research and Ethics Committee. Permission to conduct the study in respective PHCs was subsequently granted by the FCT Primary Health Care Development Board. Both written and verbal informed consent was obtained from participants. Interviews and focus group discussions were conducted privately and participants’ transcripts were pseudo-anonymised.

### Sampling

The sample for this study was drawn from both PHC workers and adult PHC clients.

For PHC clients a purposive sampling strategy was used to recruit individuals from rural and urban PHCs. Clients over 20 years old were selected on the basis of age, ethnic group and blood pressure status (i.e. hypertensive patients and clients not known to be hypertensive). Since pregnancy induced hypertension has a different course and aetiology, pregnant or lactating hypertensive females were excluded from participating in this study. With the help of the PHC co-ordinator, clients who fulfilled the inclusion criteria were recruited either for a one to one interview or for a Focus group discussion during regular clinic attendance. Those who agreed to be interviewed were interviewed at the PHC immediately. Focus group discussion participants were asked to come back to a chosen PHC on another day for the discussion. A token payment of Five Hundred Nigerian naira (approximately 1 Great Britain Pound) was given to each focus group participant to help with transportation costs.

For PHC workers, a purposive sampling strategy was also used to recruit PHC workers from all categories of PHC health workers across the PHCs in FCT. The following categories of health workers are available at PHCs in Nigeria; Medical Doctor, Nurse/Midwife, Community Health Extension Workers (CHEW), Junior Community Health Extension Workers (JCHEW), Pharmacy Technician, Laboratory Technician and Health Attendant/ Assistant [16].

With the help of the PHC coordinator and IB, PHC workers from each category were recruited for either a face to face interview or a focus group discussion over the phone. Those who agreed to be interviewed were interviewed at their respective PHC. Focus group discussion participants were asked to come to the local government headquarters office where the discussions took place. Each focus group discussion participant was given a token of five hundred naira (approximately 1 Great Britain Pound) to help with transportation costs.

### Data collection

All interviews and focus group discussions were conducted in English as all participants were able to speak English. Individual interviews lasted between 30 and 45 minutes while focus groups lasted between 1 to 2 hours. Interviews and focus groups discussions were recorded. JA conducted all interviews while JA and IB facilitated the focus group discussions.

The topic guide used in both interviews and focus group discussions was based on previous studies on beliefs about hypertension in Nigeria [7–14, 17] and earlier work on explanatory models of illness by Kleinman [18, 19, 20]. When eliciting beliefs and comparing cultural understandings of illnesses the anthropological perspective may be very helpful, therefore Kleinman’s work on explanatory models(EM) which categorizes people’s beliefs about illnesses into the cause of an illness, its course, diagnosis, symptoms and treatment was used [19, 20].

Selection of participants for interview and focus groups stopped when theoretical saturation was reached [21].

For PHC clients and PHC workers, the standard interview protocol from Kleinman and Weiss’ Explanatory Model Interview Catalogue [19, 20] composed of eight questions was used verbatim for the interviews and focus group discussion.

Probes and checks such as “tell me more”, “could you give me examples…”, “it sounds like you were saying…” and so on were used when needed to increase the richness and depth of responses. JA recorded reflections and iterative modifications about the interviews and focus group discussions in a diary and all participants were informed about the use of a reflective diary.

### Data analysis

All interviews and focus group discussion were transcribed verbatim. The transcribed data and the reflective diary formed the data used in this analysis. The data were analysed thematically using the “thematic framework approach” with the NVivo 11 software. The analysis was done concurrently with the interviews and focus group discussions so as to allow emerging themes from preceding interviews inform later interviews and focus group discussions and to determine point of saturation [21]. However, analysis was carried out separately for focus group discussion and interviews allowing convergence of recurrent themes around hypertension to be captured thereby ensuring trustworthiness of findings [21].The process of analysis was as follows:

**Familiarization with the raw data**: After all transcripts from focus group discussion and interviews were retrieved from the transcription service, JA read through the entire transcript several times while listening to the audio-tapes to ensure accuracy of transcription. This allowed JA to familiarize with the raw data and identify emerging concepts from the data.

**Identifying a thematic framework**: JA identified recurring themes from the data to form an index of data that was used to label the transcript and divide them into sections corresponding to established ideas and themes. This formed the backbone of the thematic framework.

**Coding the transcript according to themes identified:** JA indexed the transcript and highlighted them according to established ideas and themes using the NVivo 11 software.

**Charting the data**: the NVivo 11 software was used to chart highlighted portions of data under appropriate thematic framework to which they belong.

**Mapping and interpreting the data**: The charted data were examined for relationships and interpreted to obtain conclusions.

The initial analysis was done by JA. A sample of the data was independently analysed by RM and similar themes were identified. A final interpretation was agreed upon following discussion.

### Member checking

After transcription and data analysis, a sample of participants was sent a copy of the documents for their feedback. There were no requests for amendments.

## Results

### Participant characteristics

For PHC workers, a total of 41 participated in the study including 31 semi-structured in-depth individual interviews and 1 focus group discussion of 10 participants.

For PHC clients, a total of 41 participated in the study including 31 semi-structured in-depth individual interviews and 1 focus group discussion of 10 participants. One individual in-depth interview was unfortunately lost due to a corrupted audio file bringing the final sample of PHC clients to 40 participants (30 individual interviews and 10 focus group discussion participants).

In-depth interview and focus group discussion participants’ characteristics among PHC clients are described in Table 1.

**Table 1:** Characteristics of PHC clients used in the in-depth interviews and focus group discussion.

Characteristics	In-depth interview PHC clients (N = 30)	focus group discussion PHC clients (N = 10)
**Gender**		
Male	13	5
Female	17	5
**Age {Median (min, max)}in years**		
Men	35(23,67)	40(21,66)
Women	42(22,65)	40(28,53)
**Ethnicity**		
Igbo	4	2
Yoruba	3	1
Hausa	2	2
Nupe	2	3
Higi	-	1
Tarok	-	1
Etsako	2	-
Kataf	2	-
*Others	15	
**Highest education level achieved**		
Primary	2	-
Secondary	13	2
Associate degrees	2	-
University education	11	8
Post graduate	-	-
No formal education	2	-
**Blood pressure status**		
Known hypertensive	13	1
Not Known to be hypertensive	17	9

*others refer to ethnicities where there was only 1 participant of each ethnic group

In-depth interview and focus group discussion participants’ characteristics among PHC workers are described in Table 2.

**Table 2:** Characteristics of PHC workers used in the in-depth interviews and focus group discussion.

Characteristics	In depth interview PHC workers (N = 31)	focus group discussion PHC workers (N = 10)
**Gender**		
Male	17	6
Female	14	4
**Age {Median (min, max}in years**		
Men	35(29,53)	36.5(28,51)
Women	35(23,55)	28.5(25,36)
**Ethnicity**		
Igbo	4	2
Yoruba	2	1
Hausa	2	2
Nupe	4	3
Fulani	2	-
Gbagyi	2	-
Higi	2	1
Tarok	3	1
*Others	10	-
**Highest education level achieved**		
Primary	-	-
Secondary	5	2
Associate degrees	-	-
University education	24	8
Post graduate	2	-
No formal education	-	-
**Blood pressure status**		
Known hypertensive	1	1
Not Known to be hypertensive	30	9
**Health Care Worker Category**		
JCHEW	4	2
CHEW	6	2
Nurse	5	1
Medical Doctor	3	-
CHO	2	1
Health assistant	6	2
Laboratory Technician	3	1
Laboratory Scientist	1	1
Pharmacy Technician	1	-

*others refer to ethnicities where there was only 1 participant of each ethnic group

### Beliefs about hypertension

The analysis yielded four (4) themes that were shared by both clients and healthcare workers, namely (1) Meaning of hypertension (2) causes of hypertension, (3) Consequences of hypertension (4) Perceptions of treatment. However, one additional distinct theme was found among PHC workers “contextual explanation”. There were no differences in the themes that emerged from focus group discussions compared with the in-depth interviews among both PHC clients and workers.

Table 3 summarizes the similarities and differences in the themes and subthemes between PHC workers and clients.

**Table 3:** Summary of similarities and differences in themes and subthemes.

**Themes**	PHC Clients Subthemes	PHC workers Subthemes	Overall Similarities and differences
**Meaning of hypertension**	-Cultural perception -Biomedical understanding -Stress	-Biomedical understanding	All PHC workers expressed mainly biomedical understanding of hypertension. Some PHC clients shared similar beliefs with PHC workers in the area of biomedical understanding but many PHC clients had other significant cultural and psycho-social perceptions about meaning of hypertension. Overlap in ethnic understandings as well as different intra-ethnic meanings existed among PHC clients. Overall known HTN and not known HTN expressed fairly similar beliefs on meaning but differed slightly in areas such as biomedical understanding
**Causes of hypertension**	-stress -biomedical causation -drug abuse -divine destiny	-Stress -Biomedical causation -Drug abuse	Both PHC workers and clients (known HTN and not known HTN) shared mostly similar understanding of the causation of hypertension but differed in that some PHC clients believed in the role of spiritual forces.
**Consequences of hypertension**	-Poverty -Solitude -symptoms of hypertension	-illnesses caused by hypertension -symptoms of hypertension	Generally, both PHC clients and workers (known HTN and not known HTN) expressed similar beliefs about consequences of hypertension in terms of common medical symptoms of hypertension. But differed in that PHC clients did not express any complications of hypertension such as stroke as a consequence. Only PHC workers believed that it could be asymptomatic. Further, only those PHC clients who are known HTN expressed financial stress as a major consequence.
**Perception of treatment**	-cure versus manage -spiritual help -therapeutic agents -life style modification -stress management	-cure versus manage -spiritual help -therapeutic agents -Life style modification	Overall, many PHC clients and workers shared more than one similar belief about treatment for hypertension. But, most PHC clients who shared more than one belief about treatment thought that spiritual help should be an adjunct to any other treatment modality. However, only a few PHC workers would recommend spiritual help for their clients. Although most PHC clients expressed beliefs about stress management as a form of treatment, no PHC worker expressed this idea.
**Contextual explanations**			This theme was unique to PHC workers. Contextual explanation refers to the way the PHC worker would explain the diagnosis of hypertension to a client. Among all PHC workers, the explanation offered is guided by the local name and meaning of hypertension in the community in which they practice. However, each time although the local name is used explanation is tailored to reflect biomedical understanding.

### Meaning of hypertension

There were varieties of beliefs about what the term “hypertension” meant among both PHC clients and workers. This theme was divided into 3 sub-themes for PHC clients including; “Cultural perception”, “Biomedical understanding”, and “stress” and only 1 sub-theme for PHC workers; “biomedical understanding”.

Many PHC clients expressed only one understanding of hypertension. However, there were overlaps of beliefs among some PHC clients as they shared all the three understandings of hypertension elicited.

All PHC workers expressed mainly biomedical understanding of hypertension. Moreover, there was no specific difference in understanding between types of PHC worker.

Overall, on the meaning of hypertension, some PHC clients shared similar beliefs with PHC workers in the area of biomedical understanding but many PHC clients held beliefs related to culture and stress.

#### Bio-medical understanding

Some PHC clients and all PHC workers expressed understandings about meaning of hypertension related to biomedical views of raised blood pressure as measured by some medical equipment.

*“No*, *the only name I know for hypertension is hypertension when the doctor measures it with a machine” (cp4:I)*

However, PHC workers explained the meaning in more definite terms using cut-off points for abnormal blood pressure measurement. But, different cut-offs for diagnosis of hypertension were mentioned by two senior PHC workers.

Notably, during the focus group discussion for PHC workers a discussion took place over the issue of blood pressure cut-off point for diagnosis of hypertension and all arrived at a consensus that they needed to look up in the books for the acceptable cut-off point.

*“Apart from hypertension, it is also known as high blood pressure, increase in your BP that is the blood pressure is more than 160/100*, *or 160/110 that is hypertension…”(wp4:I)*

*“You see*, *when the systolic pressure rises from the range 100 to 140, that person is free. So if, the diastolic pressure too from 60–90, it is expected that, that person doesn’t have any cause for alarm so, if the systolic pressure is above 140, that is where we say the blood pressure is high now and we try to explain things to them”(wP1:I)*

All PHC clients who expressed biomedical views also expressed other views about meaning of hypertension and all were diagnosed hypertensive clients. All of those who did not express biomedical views of meaning were not known to be hypertensive.

#### Cultural perceptions

This study involved participants from twenty-one ethnic groups. Three (3) diagnosed hypertensive participants who gave the ethnic/cultural names for hypertension explained their meanings in terms of stress and biomedical related complications.

*“In Yoruba*, *we call it Ejeriru. The meaning is that, when you over labour the body, the way the heart pumps out blood, is too high and the temperature will go high and all the body will collapse, and that part of the body, will be functionless either the leg, hand or the whole body”(cp9:fg)*

*“Like in my own language*, *Igbo, we call it ‘‘ogbaramgbanyi-enu” i.e. high BP high blood pressure.It means someone who is over thinking has something disturbing or worrying him, that causes him may be, sleepless nights and all that and sometimes the person starts misbehaving at times it leads to paralysis or stroke”. (cp8:fg)*

Other ethnic understandings of hypertension elicited from this study did not relate to stress or biomedical complications but were verbatim translations of the native/ethnic/cultural names for hypertension. They include perceptions like; “the blood volume is going high”, “climbing blood”, “blood shooting up” and “a dangerous ailment that is incurable”. These perceptions were elicited from Igbo, Tiv, Hausa and Esan-edo ethnic groups respectively.

*“We call it ‘‘ogbaramgbanyi-enu” it means somebody’s blood level is going high” (cp30:I)*.

*“Hypertension…some locals call it bubun jini, but the enlightened ones would say; hawan jinni*. *It means Climbing blood (literally) but it means hypertension” (wp20:I)*.

*“Well generally in Hausa we call it hawan jini*. *It means the blood is shooting up”(cp8:I)*

*“In my dialect we have a name for it. It is called, ‘Ujagbe’. It means something that is a dangerous ailment that is un-curable”(cp21:I)*.

It was noted that among the Igbo ethnic group, different meanings were given to the same ethnic names for hypertension.

*“Like in my own language, Igbo, we call it ‘‘ogbaramgbanyi-enu” i.e. high BP high blood pressure.It means someone who is over thinking has something disturbing or worrying him, that causes him may be, sleepless nights and all that and sometimes the person starts misbehaving at times it leads to (paralysis) paralyze or stroke”*. *(cp8:fg)*

*“We call it ‘‘ogbaramgbanyi-enu” it means somebody’s blood level is going high” (cp30:I)*.

There was also overlap between the Yoruba ethnic understandings and Hausa ethnic understandings as participants belonging to these groups expressed similar meanings to different native names.

*“Well in Hausa we call it hawan jini*. *It means the blood is shooting up”(cp23:I)*

*“I don’t know any other name but the Yourba call it ejeriru which means blood shooting up”(cp28*:*I)*

All these beliefs were expressed by both diagnosed hypertensives and patients not known to be hypertensive alike. There was no indication of any distinction in beliefs between diagnosed hypertensives and participants not known to be hypertensive.

#### Stress

Some PHC clients expressed the understanding that hypertension simply meant stress. Stress was colloquially termed “thinking too much” or “thinking” which is caused by negative circumstances in life.

*“It means when you are thinking*, *like when something is disturbing you and you are thinking too much of how to get out of that problem and then that thinking enters your blood and make your blood pressure to rise”(cp10:I)*

*“It means someone who is over thinking has something disturbing or worrying him*, *that causes him may be*, *sleepless nights and all that…”(cp5*:*fg)*.

Among PHC workers, stress was only mentioned as a cause of hypertension and not understood to mean hypertension.

### Causes of hypertension

PHC workers and clients expressed similar beliefs on causes of hypertension under three subthemes namely: “stress”, “biomedical causation” and “drug abuse”. PHC clients expressed other beliefs under the subtheme “divine destiny”.

Both PHC workers and clients shared more than one understanding of the causation of hypertension. Most PHC clients who held more than one belief about causes of hypertension included stress as part of their understanding for cause of hypertension.

*“Well, they are numerous but I will just say one or two: Heredity. In your family line, your lifestyle i.e. what you eat and stress. There are also disease conditions like nephritis, diabetes; these are some of the diseases”(cp20:I)*.

#### Stress

Stress was the most prominent belief expressed in this sample as the cause of hypertension. Majority of participants (PHC workers and clients) expressed stress as the cause of hypertension. There was no specific difference in the beliefs about stress amongst diagnosed hypertensives and those not known to be hypertensive. It is also of note that there was no difference in the aetiology of stressors identified by men and women in this sample. Generally, stressors identified in this sample refer to thinking too much about life circumstances such as poverty, health related problems, relationship problems, loss of a loved one, unemployment, family responsibilities and financial demands or anything that reduces the amount of sleeping time.

*“When you are anxious about something and you are not getting it, eventually*, *it can result to hypertension” (cp7:fg)*

*“Yes if one over thinks about something in the sense that it affects your sleep. You don’t sleep well it affects the brain. Your brain doesn’t rest, neither does it get adequate rest. It can lead to high B.P”(wP2:fg)*.

One PHC client disagreed with the fact that being biologically old in age may be a risk factor for hypertension but felt that most hypertension in old age was as a result of stress in old age.

*“It depends on the person because most old people stress themselves to the extent that they will not have time to relax and some other people they have time to relax because their children do everything for them, so they don’t have anything to think about*. *It is when they start thinking about maybe the wellbeing of their children which is not okay and that might lead to hypertension”(cP29:I)*

Most PHC workers who said stress was a cause of hypertension had practical examples among their clients to back up their claims.

*“Maybe*, *all the time*, *you are busy doing this or that*, *thinking of this or that all through the night*, *you don’t sleep*, *you are having insomnia*, *it could lead to hypertension too”(wp1*:*I)*.

*“It is not just my opinion but from what I have learnt and experienced, it is caused by too much thinking, stress. People don’t have a history for rest” (wP14:I)*.

#### Bio-medical causation

Most PHC clients and all PHC workers believed that biomedical identified risk factors such as excessive salt intake, lack of exercise, excessive fat consumption, smoking and aging could be a cause of hypertension. However, for PHC workers some specifically used the word “causes” while others were more cautious preferring to use the word “predisposing factors” for hypertension.

*“I don’t think salt causes hypertension*. *It is just a predisposing i.e. a contributing factor”(wp5:fg)*

*“Smoking can make the blood pressure to increase”(wp22*:*I)*

*“Yeah because I believe that too much fat will block some blood vessels and that can cause it”(wp23*:*I)*

There were no differences found in beliefs between clients known to be hypertensive and those that are not known to be hypertensive.

However, some PHC clients who believed that biomedical risk factors could trigger hypertension disagreed with some known biomedical risk factors. Some of the disagreements were based on personal experiences or experiences of people in their social network.

*“Because as for myself I don’t sit in one place, I am always about but yet I still have it”(cp11*:*I)*

*“Why I will not agree with that is because my mother who died at the age of ninety five (95) did not have hypertension so, I don’t believe that”(cp21*:*I)*

One PHC client who didn’t agree with the fact that alcohol could be a cause of hypertension thought so because of the ability of alcohol to reduce “thinking” or stress.

*“If you take alcohol, you feel relaxed, so you don’t think”(cp31:I)*.

All PHC workers also expressed beliefs that hypertension could be genetically inherited and that it could occur as a result of complications from other medical conditions. However, this was not the case with PHC clients. Some believed it could be inherited while others did not. Most of the PHC client who expressed agreement or disagreement with heredity as a cause of hypertension did so on account of personal experiences. For most of these participants, these did not affect other held beliefs as most of them still thought that other biomedical risk factors could cause hypertension.

*“…*. *one my bosses*, *she has an eye problem and she told me that her dad had it* … *Her dad and mum had it and now one of her children is facing it*. *So likewise hypertension*, *it can be caused by heredity”(cP6*:*I)*.

*“…Like I told you earlier, that I don’t think I inherited it from my parents. Maybe, in a way, I contributed negatively either my lifestyle or diet”(cP20*:*I)*

Regarding salt intake, there were two camps in the focus group discussion. Some PHC workers specified that salt was more dangerous than some certain types of food seasoning while others disagreed.

*“I have observed that people in the North*, *hardly suffer from hypertension but in the East*, *you see a lot of adults with it because of the kind of food they eat*. *The Northerners tell me that they don’t cook with salt*, *instead they use magi in place of salt*. *You can see them using up to ten (10) to fifteen (15) cubes of magi for cooking soup but in the East*, *we don’t use maggi and some of our local foods*, *require raw salt and you know that raw salt is more dangerous than cooked salt”(wp3*:*fg)*.

*“I don’t agree with taking only maggi. As she has rightly said, the major ingredient in maggi is salt so I do not agree with taking only maggi if there is no salt, there should be no maggi too”(wP7:fg)*.

#### Drug abuse

Both PHC clients and workers expressed the idea that addiction to illicit drugs like cocaine and cannabis could cause hypertension.

*“Yes*. *In those who are involved in social vices*, *like; drugs*, *drug addict*, *someone who takes a lot of alcohol too*. *All these ones are prone to hypertension” (wP11*:*I)*.

#### Divine destiny

Two PHC clients felt that sometimes the cause of hypertension may not be attributable to any physical cause but the divine will. It is important to note that these participants still expressed beliefs about other causes of hypertension such as stress.

*“There are two types; one that comes from over-thinking and vexing, the other comes naturally from the air divinely” (cP27*:*I)*

### Consequences of hypertension

Both PHC clients and workers expressed generally similar beliefs about consequences of hypertension in terms of “symptoms of hypertension”. But PHC workers expressed other beliefs about “illnesses caused by hypertension” and PHC clients expressed other beliefs under the subtheme “solitude” and “poverty”.

#### Symptoms of hypertension

All PHC workers reported at least one symptom of hypertension. This was based on their experiences of managing patients with hypertension. The most common symptoms mentioned were headaches, palpitations, dizziness, blurred vision. Among PHC clients the commonest symptoms mentioned were palpitations and dizziness

*“I will tell the person the signs and symptoms of hypertension some are headache, dizziness and some will have palpitations”(wP15*:*I)*

A few PHC clients said they didn’t know what effects hypertension could have because they did not have the disease. All of the five participants who said this were not known to be hypertensive.

*“Well*, *since I don’t have it, I don’t think I can’t say much about it” (cP6:I)*

However, some clients not known to be hypertensive still gave their opinion on the effects of hypertension based on experiences of those in their social network.

*“Yes*, *my sister*. *Sometimes she dey say her eye dey turn (feels dizzy) and her heart dey beat fast (palpitations)*. *When she starts to feel like that*, *she has to go to the hospital to run some tests”(cP3*:*I*)

Some PHC workers also believed that hypertension could result in weight loss and sleeplessness and would tell their clients to watch out for these signs among others as mentioned above.

*“Sometimes, if you have hypertension, you lose weight, and experience headache, your blood pressure is checked or you are sent to the laboratory to know what is there”(wP18:I)*.

Only few PHC workers mentioned that hypertension could be asymptomatic. Most of those who said these were high level cadre health care workers like doctors, nurses and senior community health extension workers.

#### Illnesses caused by hypertension

Most PHC workers reported that hypertension could result in other health problems if not well managed. The most common illness reported was stroke, eye problems, hypertension in pregnancy related complications and sudden death.

*“I fear these two things; having stroke (paraplegia)” and then sudden death. Those are the things that scare me about hypertension”(wp3:fg)*.

#### Poverty

The idea of hypertension making one poor was expressed by only diagnosed hypertensive PHC clients who have been on hypertensive medications for a long time.

*“I can’t just say because if I see the situation I am in now, I can’t pay for my drugs*, *I can’t eat good food, these are the major problems I have faced”(cP24:I)*

#### Solitude

One PHC client not known to be hypertensive expressed the idea that he has observed that hypertensive patients usually like to stay in serene locations at all times as they may not want to be disturbed. It is not clear whether the participant viewed this as a positive or negative outcome. However, during the focus group discussions with PHC clients, this idea was disputed by most diagnosed hypertensive participants.

*“Well people that have hypertension, they do not like staying around places that are disturbed, they always want to be where it is quiet” (cP6:I)*.

### Perceptions of treatment

PHC workers and clients’ beliefs on treatment of hypertension could be summarised under 3 similar subthemes namely: “cure versus manage”, “spiritual help”, “life style modification” and “therapeutic agent”. But PHC clients expressed other beliefs under the subtheme “stress management”

Overall, many PHC clients and workers shared more than one belief about treatment for hypertension. Most PHC clients who shared more than one belief about treatment thought that spiritual help should be an adjunct to any other treatment modality you choose to engage with. However, only a few PHC workers would recommend spiritual help for their clients.

*“The only thing I have to tell the whole country (Nigeria) that is you and I*, *as soon as you have any problem with hypertension, come to the hospital and take care of it, let them test you, collect drugs and after then pray to God to heal you”(cP9:I)*

There was no relationship between PHC client’s status (i.e. hypertensive or not known to be hypertensive) and beliefs about treatment of hypertension. However, among PHC clients there was a relationship between beliefs about causes and appropriate treatment for hypertension as most participants who believed that stress and biomedical risk factors were causes of hypertension believed that treatment modalities should include stress management and life style modifications.

#### Cure versus manage

There was a general consensus amongst all PHC workers that hypertension can be managed and not cured. However, PHC clients’ response on whether hypertension would last for a short or long time was variable. A few believed that hypertension could be cured while many thought that it could only be managed. There was no relationship noted between the beliefs about the cause of hypertension and curability. Many who thought hypertension could be managed were influenced by personal and shared experiences.

*“I heard some people say that on its own after a while it vanishes” (cP30*:*I)*

*“You know one thing I don’t believe this thing has a remedy or a cure… (cp31*:*I)*

#### Spiritual help

Only a few PHC workers thought that spiritual forces could help in the treatment of hypertension. Most of those who prescribe spiritual help for their clients specifically recommend it as means of alleviating negative life circumstances that can lead to thinking too much and in turn hypertension. There was no relationship between health worker cadre and recommendation of spiritual help.

*“Any issue you know you cannot handle*, *don’t think about it but pray and hand it over to God” (wP9:I)*

However, although most PHC clients believed spiritual help was useful, they all thought it should be combined with other treatment modalities. However, one hypertensive female PHC client believed that God would heal everybody in the future.

*“God will heal everybody someday”(cP17*:*I)*

#### Therapeutic agents

Therapeutic agents refer to beliefs about the use of some external agents such as orthodox drugs or herbal medications. Most PHC workers believed that you needed some form of therapeutic agent to bring down blood pressure. Orthodox medications were believed to be effective if strictly adhered to. As for herbal medications while majority will not recommend it, a few PHC workers would still prescribe it for their clients. In addition, most PHC workers would not recommend taking herbal medications together with orthodox medication as it could cause adverse reactions. Those who prescribe herbal medications do it based on positive experiences in the past. One PHC worker said:

*“I used to be one of those that is totally against herbal medication but I just noticed of recent*, *on the herbal medication that was given to a hypertensive patient with a case of obesity and I think they were just natural roots given to her to keep boiling and drinking. Of a truth, she did as she was told and she involved herself in exercise as well. The obese nature dropped drastically which the patient continued taking those herbs for almost three weeks or so and it dropped and the person continued with the exercise….” (wP2:Fg)*

However, half of PHC clients believed that orthodox drugs alone as prescribed by a doctor was effective in treating hypertension but only a few believed that using herbal medications alone could be effective. Some of the participants who believe that herbal medications alone could be effective thought that you needed a doctor to make a diagnosis before using herbal medications.

*“You can take herbs when you know what the problem is. When you know what you are having it is then you can start taking herbs instead of taking anything when you don’t know the problem”(cP2;I)*.

A few PHC clients believed that you could use both herbal and orthodox medications together.

*“Yes they should take both*. *You can use the herbal for a start then the orthodox medicine”(cP7:I)*

#### Life style modification

All of the PHC workers and most PHC clients believed that life style modification was a key element in the treatment of hypertension.

*“Yes, number one, you have to assure them; two, you have to sensitize them .i.e. you do community health education and mobilization, and that they should be able to take their drugs constantly” (wP12:I)*.

*“The kind of food you eat matters a lot because it goes into the body so*, *if you take in too much salt, fats, can lead to it. You will reduce some kind of food intake that is what I think apart from the drugs”(cP29:I)*

However, only about half of the PHC workers reminded their patients regularly about modification of life style habits during consultation.

#### Stress management

Since majority of PHC clients thought that stress was a cause of hypertension, many thought that stress management i.e. not thinking too much and taking a rest was a personal remedy for hypertension. However, the idea of stress management was not particularly expressed by PHC workers of any cadre.

*“First I would call it a personal remedy. You should try and reduce your thinking, then maybe stay away from things that cause you to think, stay away from problems although you I don’t think you can escape problems but you can just try to help yourself then constant check-up”(cP7:I)*.

### Contextual explanation

Contextual explanation refers to the way the PHC worker would explain the diagnosis of hypertension to a client. Among all PHC workers, the explanation offered is guided by the local name and meaning of hypertension in the community in which they practice. However, each time although the local name is used explanation is tailored to reflect biomedical understanding.

*“Where I practice*, *I have Hausas mixed with Yoruba’s, when they come; the language we use in Yoruba is Ejeriru then for the Hausas, Hawan Jini. Those are the languages we use in our practice. When we meet someone who is advanced in knowledge, we use hypertension. These are the two languages use in the place where I work”.(wp6:I)*

*“It depends. If it is in English, and for lay persons that are a bit literate, they don’t know hypertension, so you tell them, high blood pressure or if it is in the area council, which is multi-lingual. It has so many tribes. The commonest I see, they have Hausa, Igbirras and Ganagana. Hausa call it Hawan Jini, Igbirra call it “anyahire”, i.e. blood has risen or raised blood. I can’t remember how the Ganana call it but it has to do with blood too. I am talking about how blood has risen it is just a sentence and not a word” (wP24: I)*.

## Discussion

In this study we elicited beliefs about hypertension among PHC clients and workers across PHCs in the Federal Capital Territory of Nigeria. PHC clients and workers expressed beliefs which were grouped into 4 themes—(1) Meaning of hypertension (2) causes of hypertension, (3) Consequences of hypertension (4) Perceptions of treatment. PHC workers expressed beliefs grouped into a unique theme—(5) “contextual explanation”. In-depth interviews and focus group discussions yielded a wide variety of insights into similarities and differences in beliefs between PHC workers and clients. This study is first of its kind in Nigeria highlighting the beliefs of both PHC clients and workers about hypertension.

Regarding the meaning of hypertension, while all of PHC workers’ perspectives centred on biomedical definitions of the disease, most PHC clients expressed ideas around cultural explanations and social aspects of life. Lay beliefs on the understanding of hypertension in terms of social related stressors are not new. Similar findings have been shown in studies on beliefs of hypertension in Nigeria [13, 14, 22] and in other places [23–29]. However, description of meaning hypertension among Nigerians in terms of cultural understanding is relatively new and was only recently described in a similar study conducted by the authors on Nigerian immigrants to the United Kingdom [17]. Interestingly, new cultural meanings such as- hypertension meaning a “disease that is in-curable and dangerous”- different from those highlighted in Akinlua et al 2017 [17] were elicited in this study.

In agreement with the findings from Akinlua et al 2017 [17], some PHC clients also held multiple beliefs about meaning of HTN at the same time. However, there was no evidence of a major overriding belief among those who had multiple understandings. Although this has not been reported in other studies, it is pertinent that PHC workers understand this fact to guide counselling sessions appropriately. Interestingly, the study showed that PHC workers were already contextualizing the explanation of HTN in relation to cultural understanding.

In this study, proven biomedical risk factors such as smoking, excessive alcohol intake, inadequate exercise, excessive fat and salt intake and stress and drug abuse were believed by both PHC workers and PHC clients to be major causes of hypertension. This is consistent with other studies on Nigerians [13, 14, 22] and in other parts of the world [23–29]. However, the study by Beune et al [23] showed that beliefs about causes of hypertension differed between health workers and lay people with hypertension. Notably, among some PHC workers, the idea that food seasoning such as *Maggi* is not salt and that cooked salt is less harmful than uncooked salt has not been reported in other studies. Also in agreement with other studies conducted on lay Nigerians [13, 14, 17] very few PHC clients thought that HTN could be inherited, but heredity was a key factor in some studies on African-Americans [24, 28].

Moreover, disagreement between cut-off points for hypertension among health workers is not new as this has been demonstrated in other studies [30,31]. This is probably because of use of different guidelines among health workers. It is imperative that unified guidelines are used in primary care settings in Nigeria so as to ensure accurate risk assessment and prompt commencement of treatment when needed.

Further, regarding the consequences of hypertension, it is important to note that no PHC client thought that it could be asymptomatic. This was also reported in the studies among Nigerian immigrants in the UK [17]. This finding is quite significant in that it may affect health seeking behaviour among lay people. As such this needs to be addressed in health promotion programs.

There was ambiguity over perceived symptoms of hypertension even among diagnosed hypertensives PHC clients. Among diagnosed hypertensives, the prohibitive cost of drugs and unavailability of drugs resulted in poor adherence and impoverished them. This has been reported in other studies conducted on Nigerians [7–11,13,14] and elsewhere [24,26,28,32]. Interestingly, unlike PHC workers, PHC clients did not express ideas of disease complications from hypertension. This is in contrast to other studies conducted on Nigerians [14, 17].

Between PHC clients and PHC workers, it was possible to summarize beliefs about treatment of hypertension under similar sub-themes with slight differences between them.

### Study strengths and limitations

This study is first of its kind in Nigeria highlighting the beliefs of both PHC clients and workers about hypertension. It explored the beliefs of hypertension among both PHC clients and PHC workers across a wide range of ethnic groups in Nigeria.

The background of JA who is medical doctor with a Yoruba ethnic Nigerian background who has worked in the Nigerian primary care may have influenced the interpretation of the data. JA had also conducted similar research among highly educated Nigerian immigrants in the UK, which may have influenced the interpretation. However, the immersion in that data may have facilitated both the elucidation of beliefs from participants in this study as well as influencing the interpretation of the data. JA conducted all the interviews but JA and IB moderated the focus group discussions and the interpretation of the data was discussed with other members of the research team and a final thematic framework agreed upon.

Member checking was also done. Although focus groups discussion have limited value in exploring complex beliefs, semi-structured in-depth interviews which are best suited for this purpose was also used. The use of these two different methods of data collection also ensured triangulation.

Although some clients in rural areas may not be able to speak English, all the participants of the study were able to speak English. This may have meant that we didn't access beliefs held by those that didn't speak English.

As it is commonly experienced in focus group discussions, some participants were very vocal while others were more passive. To ensure the ideas of everyone was captured, vocal participants were encouraged to give others a chance to talk first.

Only two focus groups were conducted due to limited resources and time constraints.

### Implications

This study highlights important similarities and differences in beliefs about hypertension among PHC clients and PHC workers that have significant implications for management of hypertension in primary care settings in Nigeria.

Although PHC clients and PHC workers had areas where they differed, it is evident that there are many areas where beliefs about hypertension were similar. PHC clients not known to have hypertension tend to generally have similar beliefs with those diagnosed with hypertension except that only those who live with the disease were able to explain certain consequences such as financial stress of treatment. Understanding these similarities and differences in beliefs is important to developing a shared understanding of the condition that is necessary to develop good practice at the client-healthcare worker level.

Specifically, the act of contextual explanation of hypertension by PHC workers should be encouraged. In addition, cultural competence training should be included as part of the curriculum for healthcare workers, especially in the non-physician healthcare worker training program as they form more than 90% of the PHC workforce in Nigeria.

Furthermore, health program designers should design programs for health care workers that reinforce the attitude of working with the beliefs that are present in any culture to develop a constructive therapeutic relationship.

Also, to ensure uniformity and comparability in future research data from PHCs in Nigeria, it is important that the next review of the manual for operations of PHCs in Nigeria emphasizes a uniform blood pressure cut-off.

## Conclusion

Eliciting beliefs and experiences among service users and service providers is very important as this could help identify important areas that need change and ultimately result in improvement of service provision. Given that beliefs are constructs of culture, environment and personal experiences, this study identifies many beliefs about hypertension that are both amenable to change and more importantly facilitates shared understandings necessary for achieving success in the control of hypertension at the individual and population level.
